# The prevalence and real‐world therapeutic analysis of Chinese patients with KRAS‐Mutant Non‐Small Cell lung cancer

**DOI:** 10.1002/cam4.4739

**Published:** 2022-04-08

**Authors:** Hanxiao Chen, Dingzhi Huang, Gen Lin, Xue Yang, Minglei Zhuo, Yujia Chi, Xiaoyu Zhai, Bo Jia, Jingjing Wang, Yuyan Wang, Jianjie Li, Tongtong An, Meina Wu, Ziping Wang, Jun Zhao

**Affiliations:** ^1^ Key Laboratory of Carcinogenesis and Translational Research (Ministry of Education/Beijing), Departments of Thoracic Medical Oncology Peking University Cancer Hospital and Institute Beijing China; ^2^ Department of Thoracic Oncology, Key Laboratory of Cancer Prevention and Therapy, Tianjin Medical University Cancer Institute & Hospital, National Clinical Research Center for Cancer, Tianjin Lung Cancer Center, Tianjin Cancer Institute and Hospital Tianjin Medical University Tianjin China; ^3^ Departments of Thoracic Oncology, Fujian Cancer Hospital Fujian Medical University Cancer Hospital Fuzhou China

**Keywords:** efficacy, KRAS mutation, NSCLC, prevalence, treatme

## Abstract

**Objective:**

Kirsten rat sarcoma viral oncogene homolog (KRAS) is an important driver gene of non‐small cell lung cancer (NSCLC). Despite a rapid progress achieved in the targeted therapy, chemotherapy remains the standard treatment option for patients with KRAS‐mutant NSCLC. This study aimed to assess real‐world data of Chinese patients with KRAS‐mutant NSCLC undergoing chemotherapy and/or immunotherapy.

**Methods:**

KRAS mutational status was analyzed using next‐generation sequencing of 150,327 NSCLC patients from the Lung Cancer Big Data Precise Treatment Collaboration Group (LANDSCAPE) project (Cohort I). Treatment data were collected and analyzed retrospectively from 4348 NSCLC patients who were admitted to the Peking University Cancer Hospital and Institute between January 2009 and October 2020 (Cohort II).

**Results:**

In Cohort I, 18,224 patients were detected with KRAS mutations (12.1%) of whom G12C (29.6%) was the most frequent subtype, followed by G12D (18.1%) and G12V (17.5%). In case of concomitant mutations, TP53 had the highest incidence of 33.6%, followed by EGFR (11.6%), STK11 (10.4%), KEAP1(6.2%), and CDKN2A (6.0%). Cohort II included 497 patients (11.4%) with KRAS mutations. In the first‐line chemotherapeutic analysis of Cohort II, patients benefited more from the pemetrexed/platinum (PP) regimen than the gemcitabine/platinum (GP) or taxanes/platinum (TP) regimen (median progression‐free survival [PFS], 6.4 vs. 4.9 vs. 5.6 months, hazard ratio [HR] = 0.65, 95% confidence interval [CI] 0.48–0.88, *p* = 0.033 and HR = 0.69, 95% CI 0.47–1.00, *p* = 0.05, respectively), with no significant difference when combined with bevacizumab. Regarding patients who received immune checkpoint inhibitors (ICIs), the objective response rate was 26% for a median PFS of 9.6 months (95% CI 6.16–13.03). Patients who received ICIs combined with chemotherapy had a significantly longer survival than monotherapy (median PFS, 13.9 vs. 5.2 months, HR = 0.59, 95% CI 0.35–0.99, *p* = 0.049).

**Conclusion:**

KRAS is an important driver gene in NSCLC, compromising 12.1% in this study, and G12C was noted as the most common subtype. Patients with KRAS‐mutant NSCLC could benefit from pemetrexed‐based chemotherapy and ICIs.

## Graphical abstract

This was the first study to analyze the treatment of KRAS‐mutant NSCLC patients in China, including chemotherapy and immunotherapy. Overall, the rate of KRAS mutation was approximately 11.4%‐12.1% in the Chinese patients with KRAS‐mutant NSCLC, and G12C was found as the most common subtype. In chemotherapy, patients with KRAS mutations could benefit more from the pemetrexed‐based regimen, regardless of mutational sites. ICIs may be a fruitful treatment modality for patients with KRAS‐mutant NSCLC, especially combined with chemotherapy.

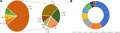

## 1 INTRODUCTION

Lung cancer is one of the leading causes of cancer‐related mortality worldwide.^1^ Owing to a great progress achieved in the targeted therapy of advanced non‐small cell lung carcinoma (NSCLC) patients, harboring candidate driver genes, including epidermal growth factor receptor (EGFR) mutations, and ALK and ROS1 rearrangements, the overall survival (OS) rate has been markedly prolonged.^2^, ^3^ Meanwhile, other patients without opportunities for target therapy are still fighting for their lives, especially individuals with KRAS mutations who were previously thought to be insensitive to chemotherapy with poor prognosis, accounting for 5%–10% and 25%–50% of NSCLC cases in the Chinese population and 25%–50% in Western countries, respectively.^4^, ^5^, ^6^, ^7^, ^8^

Kirsten rat sarcoma viral oncogene homolog (KRAS) is a member of the Ras family of proteins, which function as signal transducers between cell membrane‐based growth factor signaling and the mitogen‐activated protein kinase (MAPK) pathways. RAS can convert a molecule called active guanosine triphosphate (GTP) into inactive guanosine diphosphate (GDP).^5^ When mutations occur in KRAS, GTPase activity is inhibited, leading to the activation of the Ras protein and downstream signaling pathways.^9^, ^10^ Generally, KRAS mutations, influencing exons 2 and 3, are the most common, with G to C transition in codons 12 or 13, resulting in G12C mutations (33.6%), followed by G12D (23.9%), G12V (22.1%), and G12A (7.1%) mutations.^11^ Besides, KRAS mutations were reported to be mutually exclusive with EGFR mutations and EML4‐ALK translocations. However, other genes, mainly tumor suppressor genes, significantly co‐mutate with KRAS, including TP53 (42%), STK11 (29%), and KEAP1/NFE2L2 (27%).^12^ The high‐diversity of KRAS mutation subtypes and concurrent mutations of the above‐mentioned genes may associate with a poor response to anticancer therapy. However, whether KRAS mutations could be defined as a prognostic or predictive factor of chemotherapy in NSCLC remains controversial, owing to the existence of a noticeable heterogeneity among previous studies.^7^, ^8^, ^13^

In recent years, several scholars have attempted to target KRAS and the downstream signaling pathways, including the development of KRAS inhibitors (Sotorasib and MRTX 849),^14^, ^15^ MEK inhibitors (selumetinib, trametinib, etc.), and focal adhesion kinase (FAK) inhibitors.^16^, ^17^, ^18^ The United States Food and Drug Administration (FDA) has approved sotorasib, a first‐in‐class KRAS‐G12C inhibitor, for NSCLC. In an open‐label phase 1/2 study of sotorasib, 124 evaluable NSCLC patients with KRAS G12C mutations were treated with sotorasib (960  mg), 81% of whom had previously received platinum‐based chemotherapy and PD1/L1 inhibitors, in which objective response rate (ORR) of 37.1%, disease control rate (DCR) of 80.6%, median progression‐free survival (PFS) of 6.8  months, and median OS of 12.5  months could be achieved, demonstrating significant clinical benefits of sotorasib.^15^ With the wide application of immune checkpoint inhibitors (ICIs) in the treatment of NSCLC, programmed cell death protein 1 (PD‐1), and programmed death‐ligand 1 (PD‐L1) antibodies have been reported to improve survival to varying degrees in NSCLC patients with KRAS mutations,^19^, ^20^ both chemo‐naïve and chemorefractory. Despite a rapid progress attained in the development of new drugs, chemotherapy and/or immunotherapy remains the standard first‐line treatment strategy for NSCLC patients with KRAS mutations. However, which chemotherapy regimens are more effective and which patients would benefit more are open questions. In the present study, we aimed to analyze various treatments administered to Chinese patients with KRAS‐mutant NSCLC, including first‐line chemotherapy and/or immunotherapy.

## 2 METHODS

### 2.1 Patients

**FIGURE 1:**
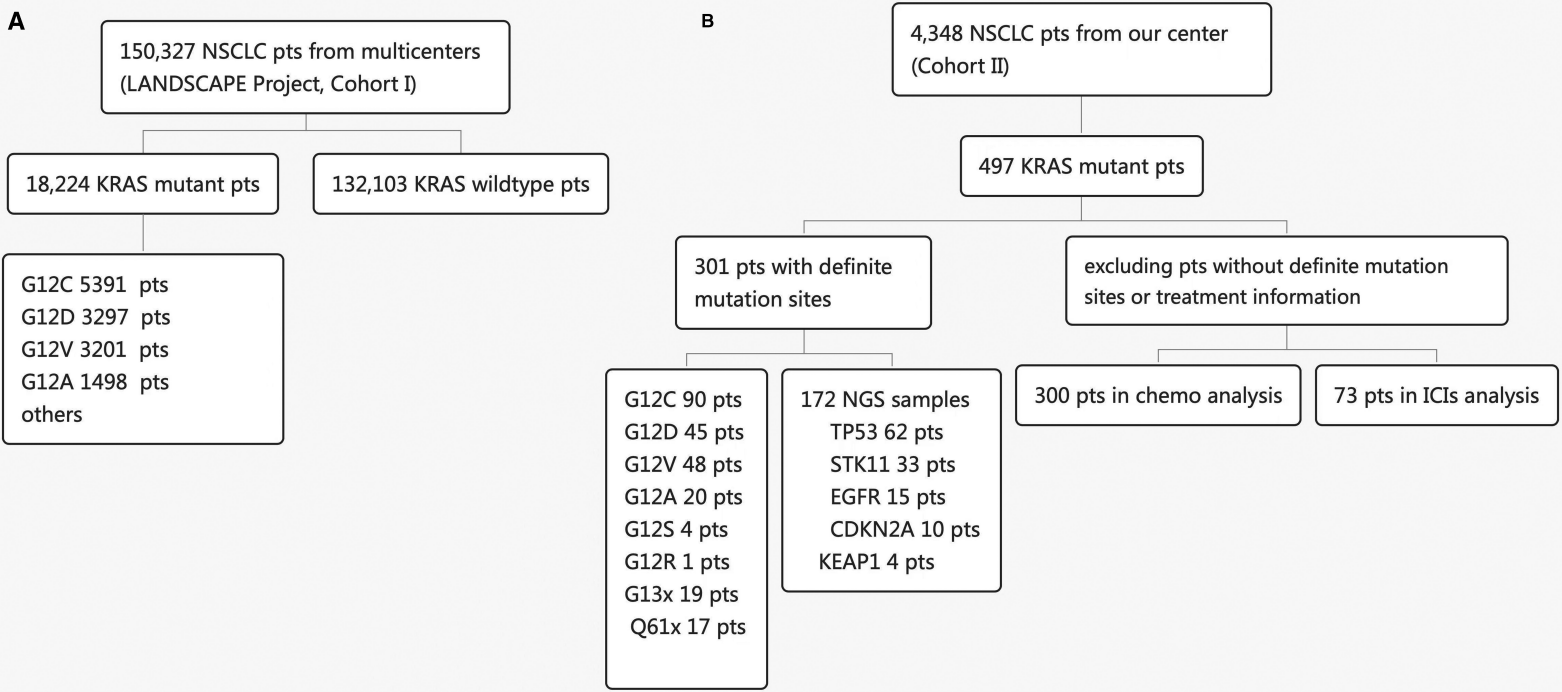
Flow charts of patients with KRAS‐mutant NSCLC enrollment in the study. (A) Patients included from the Lung Cancer Big Data Precise Treatment Collaboration Group (LANDSCAPE) project (cohort I). (B) Patients included and excluded from the Department of Thoracic Medical Oncology, Peking University Cancer Hospital and Institute (Cohort II). Pts means patients. KRAS, Kirsten rat sarcoma viral oncogene homolog; NSCLC, non‐small cell lung cancer

We retrospectively analyzed the next‐generation sequencing (NGS) data of the 150,327 NSCLC cases from the Lung Cancer Big Data Precise Treatment Collaboration Group (LANDSCAPE) project (Cohort I), mainly provided by 10 Chinese genetic testing institutions, and analyzed the incidence of KRAS and the different subtypes (Figure 1A).

We also pathologically diagnosed 4348 patients with NSCLC in the Department of Thoracic Medical Oncology, Peking University Cancer Hospital and Institute (Beijing, China) between January 2009 and October 2020 (Cohort II) (Figure 1B). The clinicopathological data of patients with KRAS‐mutant NSCLC were retrospectively analyzed, including age, sex, smoking status, histological type, pathological stage, molecular information, treatment regimen, and clinical outcomes. Tumor‐node‐metastasis (TNM) stage was determined according to the seventh edition of the Lung Cancer Staging System proposed by the American Joint Committee on Cancer. Tumor response was evaluated according to the Response Evaluation Criteria in Solid Tumors (RECIST, version 1.1) every 6–9  weeks. The study was approved by the Ethics Committee of Beijing Cancer Hospital (Beijing, China; Approval No. 2021KT33).

### 2.2 Detection of KRAS mutations

Tumor tissue and/or blood samples (10  ml) were collected before systemic treatment. Different methods were used for detection of KRAS mutations. Denaturing high‐performance liquid chromatography (DHPLC) was performed from 2009 to 2014, as described previously.^21^ Fluorescence polymerase chain reaction (PCR)‐based AmoyDx® KRAS Mutation Detection Kit (Amoy Diagnostics Co., Ltd.) was utilized to identify KRAS mutations, according to the manufacturer's instructions. NGS was adopted for the testing of multiple genes.

### 2.3 Statistical analysis

Categorical variables were expressed as frequency (count) or percentage. Quantitative variables were described as median (interquartile range). The categorical variables were analyzed using the chi‐square test. Kaplan–Meier curve analysis and the log‐rank test were employed to carry out survival analysis. In addition, PFS was defined as the time from the first day of treatment to the disease progression, death, or the last day of follow‐up. A multivariate Cox proportional‐hazards regression model was used with potential risk factors as covariates, and hazard ratio (HR) and 95% confidence intervals (95% CIs) were determined. A two‐sided *p* < 0.05 was considered statistically significant. The statistical analysis was undertaken using the SPSS 26.0 software (IBM Corp.).

## 3 RESULTS

### 3.1 Frequency and the subtypes of KRAS mutations

**FIGURE 2:**
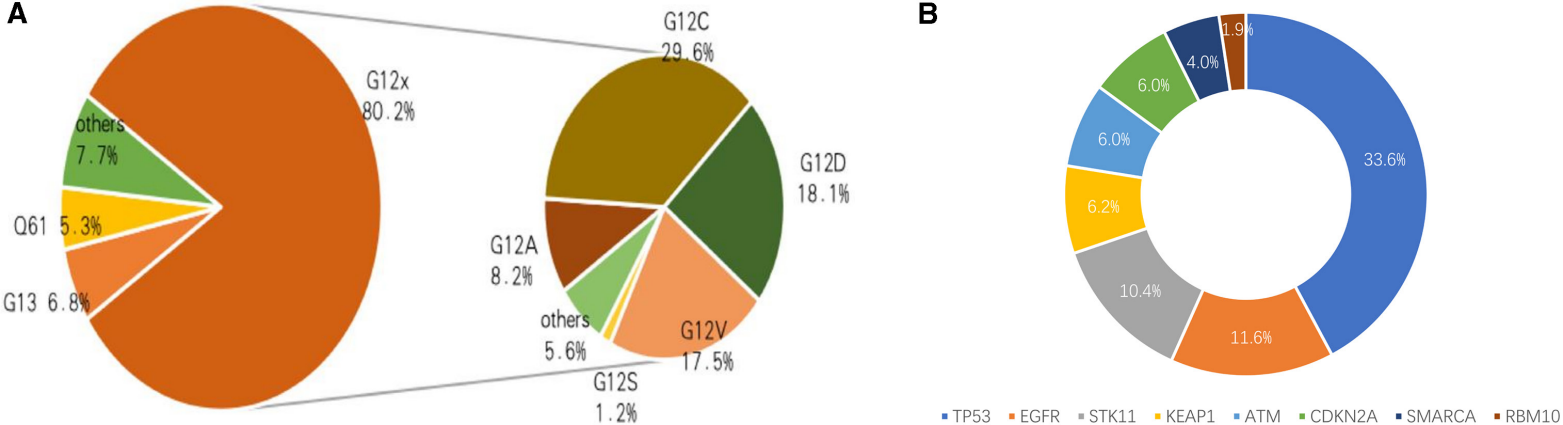
The subtypes of KRAS mutants and the incidence of co‐mutantions in Cohort I. (A) Pie charts of patients with KRAS‐mutant NSCLC and the proportions of different subtypes of KRAS mutations in Cohort I. (B) Incidence of co‐mutations in patients with KRAS‐mutant NSCLC. KRAS, Kirsten rat sarcoma viral oncogene homolog; NSCLC, non‐small cell lung cancer

In Cohort I, 18,224 NSCLC patients were detected with KRAS mutations, and the frequency of KRAS mutations was 12.1% (Figure 1A). The frequency of KRAS mutations in lung adenocarcinoma and squamous cell lung carcinoma was 12.8% and 8.0%, respectively. Among the subtypes, the incidence of G12C was the highest (29.6%), followed by G12D (18.1%), G12V (17.5%), and G12A (8.2%) (Figure 2A). In case of concomitant mutations, TP53 had the highest incidence of 33.6%, followed by EGFR (11.6%), STK11 (10.4%), KEAP1(6.2%), and CDKN2A (6.0%) (Figure 2B).

In Cohort II, a total of 497 (11.4%) patients were diagnosed with KRAS‐mutant NSCLC. G12C (40%) was the most frequent subtype detected, followed by G12V (18%), G12D (17%), and G12A (10%) (Figure 1B). Other types of mutations included G12R/S, G13C/D, Q61H/K/L, A59T/G, K117N, and A146V/T. Multiple sites of mutations were simultaneously detected in three patients: A59T/Q61K, G12C/A146T, and G12C/G12D. Among 172 samples that were analyzed by NGS, co‐mutations were detected in 110 samples, including TP53 (62, 36.0%), STK11 (33, 19.2%), EGFR (15, 8.7%), CDKN2A (10, 5.8%), and KEAP1(4, 2.3%).

### 3.2 Patients' demographic and clinical characteristics

In the Cohort II, men accounted for the majority (72.8%) of the KRAS mutations. The patients' median age was 62 (range, 27–86) years old. A total of 411 (82.9%) cases were diagnosed with lung adenocarcinoma, 45 (9.1%) with squamous cell lung cancer, and 40 with other types of lung cancer (large cell lung cancer, sarcomatoid carcinoma, and carcinoma NOS [not otherwise specified]). KRAS mutations were detected by DHPLC (*n*  =  149), PCR (*n*  =  176), and NGS (*n*  =  172). The patients' clinicopathological data are listed in Table 1.

**TABLE 1:** Clinicopathological characteristics of patients with KRAS‐mutant NSCLC

Characteristics	Cohort II (*N*/%)
Age (years old)	
<60	197 (41.4)
≥60	279 (58.6)
Sex	
Male	362 (72.8)
Female	135 (27.2)
Smoking history	
Current/former	269 (61.7)
No	167 (38.3)
Histology	
Lung adenocarcinoma	411 (82.9)
Squamous cell lung cancer	45 (9.1)
Others	40 (8.1)
Stage (AJCC 8th)	
I	31 (6.6)
II	14 (3.0)
III	82 (17.5)
IV	341 (72.9)
Mutational site	
G12	263 (74.7)
G13	19 (5.3)
Others	71 (20)
Co‐mutations	
TP53	62 (36.0)
STK11	33 (19.2)
CDKN2A	10 (5.8)
EGFR	15 (8.7)
First‐line chemotherapy	
Pemetrexed based	220 (65.7)
Gemcitabine based	67 (20.0)
Taxanes based	41 (12.2)
Bevacizumab combined with chemotherapy	
Yes	64 (19.1)
No	271 (80.9)
Immunotherapy	
Single	45 (58.4)
Chemotherapy combined	23 (29.9)
Angiogenesis TKIs combined	9 (11.7)

Abbreviations: AJCC, American Joint Committee on Cancer; CDKN2A, cyclin‐dependent kinase inhibitor 2A; EGFR, epidermal growth factor receptor; KRAS, Kirsten rat sarcoma viral oncogene homolog; STK11, serine/threonine kinase 11; TKIs, tyrosine kinase inhibitors; TP53, tumor protein p53.

Totally, 335 patients received first‐line chemotherapy, of whom 64 (19.1%) cases were concurrently treated with bevacizumab. The major chemotherapeutic regimens are presented in Table 1. Besides, 77 patients underwent immunotherapy, single drug or combination treatment (Table 1).

### 3.3 Response to first‐line chemotherapy and survival analysis

A total of 300 patients who received first‐line chemotherapy with evaluable data were divided into three groups, including the PP (pemetrexed/platinum, *n*  =  198), GP (gemcitabine/platinum, *n*  =  64), and TP (taxane/platinum, *n*  =  38) groups. Two hundred and thirty‐nine patients received chemotherapy without bevacizumab, concerning therapeutic efficacy, there was no significant difference among the three regimens, with an ORR of 26.5% for the TP group(*n*  =  34), 25.5% for the PP group (*n*  =  145), and 23.3% for the GP group (*n*  =  60). In addition, there were no statistically significant differences in DCR, as shown in Table 2. Besides, 64 patients were treated with bevacizumab simultaneously, which did not indicate improved efficacy (50%, 50%, and 26.4% in the TP [*n*  =  4], GP [*n*  =  4], and PP [*n*  =  53] groups, respectively; *p*  =  0.43, 0.42, and 0.33, respectively). We also compared clinical efficacy for the most common subtypes of KRAS mutations. Although numerically the G12C group accounted for the highest clinical efficacy (ORR  =  31.7%), there was no statistically significant difference among the three mutation subtypes (Table 2).

**TABLE 2:** Associations among first‐line chemotherapy regimens, mutated sites of KRAS, and treatment response

	ORR (%)	DCR (%)	*p*‐value
Regimens combined without beva			0.95
Pemetrexed + platinum	25.5 (37/145)	75.8 (110/145)	
Gemcitabine + platinum	23.3 (14/60)	78.3 (47/60)	
Taxanes + platinum	26.5 (9/34)	71.4 (25/34)	
Regimens combined with beva			0.17
Pemetrexed + platinum + beva	26.4 (14/53)	86.8 (46/53)	
Gemcitabine + platinum + beva	50 (2/4)	75 (3/4)	
Taxanes + platinum + beva	50 (2/4)	100 (4/4)	
Mutated sites of KRAS			0.72
G12C	31.7 (20/63)	85.7 (54/63)	
G12D	20.0 (7/35)	82.9 (29/35)	
G12V	22.6 (7/31)	81.6 (25/31)	

*Note*: beva = bevacizumab.

Abbreviations: DCR, disease control rate; KRAS, Kirsten rat sarcoma viral oncogene homolog; ORR, overall response rate.

**FIGURE 3:**
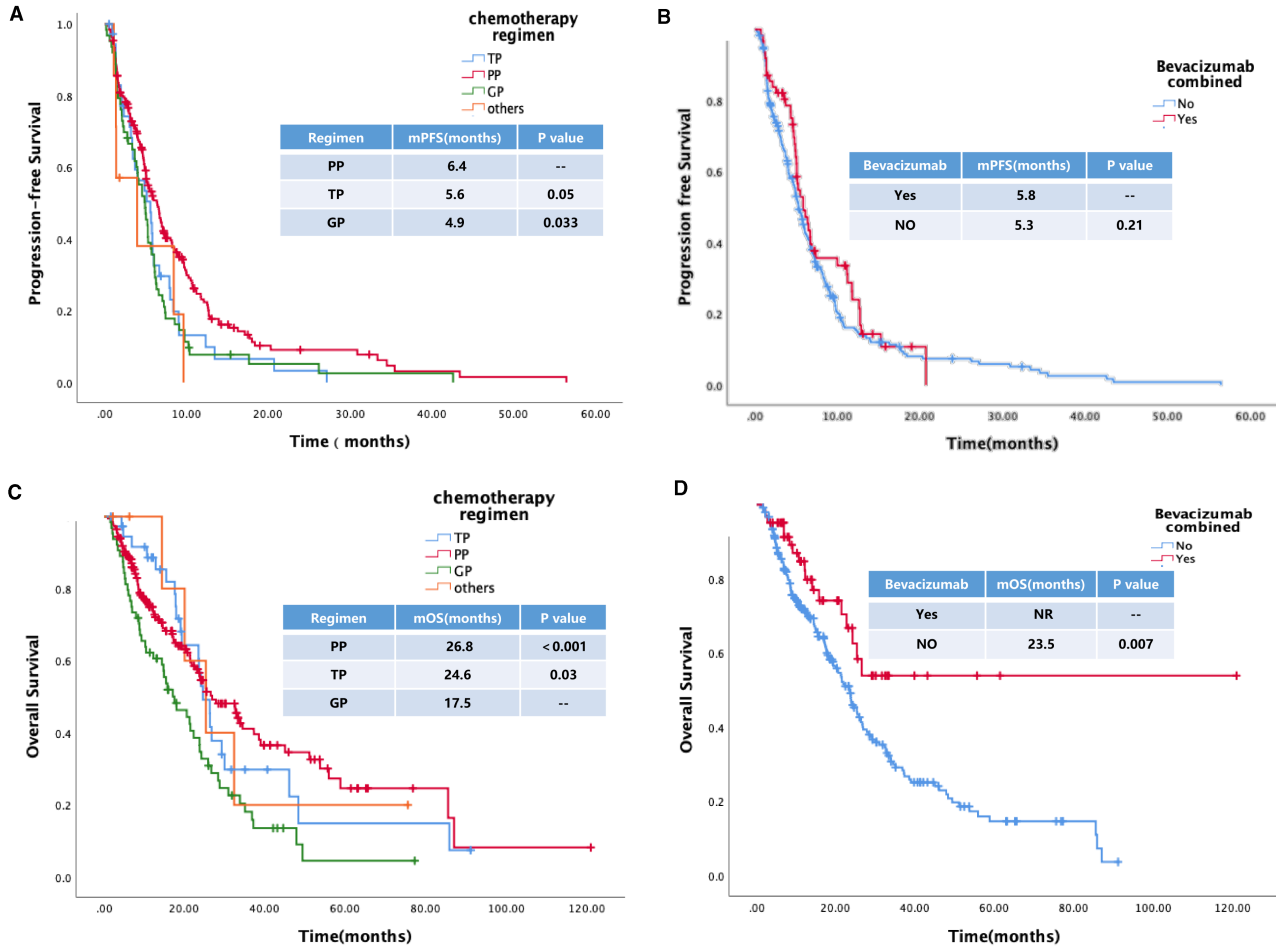
Survival of patients with KRAS‐mutant NSCLC who were treated with chemotherapy as first‐line therapy. (A) PFS of patients receiving different chemotherapy regimens. Patients benefited more from the PP regimen than the GP or TP regimen (6.4 vs. 4.9 vs. 5.6 months; *p* = 0.033 and 0.05, respectively). (B) Combined with bevacizumab did not improve median PFS (5.8 and 5.3 months, respectively; HR = 0.82, 95% CI 0.59–1.13, *p* = 0.21). (C) Survival benefit of patients receiving GP regimen was significantly inferior to that of TP and PP regimens (17.5 vs. 24.6 vs. 26.8 months, *p* = 0.03 and *p* < 0.001, respectively). (D) Combined with bevacizumab improved median OS (HR = 0.49, 95% CI 0.30–0.82, *p* = 0.007). CI, confidence interval; GP, gemcitabine/platinum; HR, hazard ratio; KRAS, Kirsten rat sarcoma viral oncogene homolog; NSCLC, non‐small cell lung cancer; PFS, progression‐free survival; PP, pemetrexed/platinum; TP, taxanes/platinum

Median PFS after the first‐line platinum‐based chemotherapy was 5.5  months (95% CI 4.9–6.1). There was no significant difference between lung adenocarcinoma and other pathological subtypes (5.8 and 4.9  months; HR  =  1.25, 95% CI 0.99–1.57, *p*  =  0.06). Compared with other regimens, we found that patients benefited more from the PP regimen (6.4  months) than the GP (4.9  months; HR  =  0.65, 95% CI 0.48–0.88, *p*  =  0.033) or TP (5.6  months; HR  =  0.69, 95% CI 0.47–1.00, *p*  =  0.05, Figure 3A) regimen. Combined with bevacizumab, PFS did not significantly improve (5.8 and 5.3  months, respectively; HR  =  0.82, 95% CI 0.59–1.13, *p*  =  0.21; Figure 3B). In addition, PFS was assessed in the G12x and G13x subtypes, suggesting that there were no significant differences among different exons. Similarly, among the most common three subtypes, no significant differences were detected in PFS, with a median PFS of 5.7, 6.6, and 6.6  months in G12C, G12D, and G12V groups, respectively (HR  =  1.09, 95% CI 0.86–1.39, *p*  =  0.49). In the multivariate logistic regression analysis, only chemotherapy regimen was the factor that influenced the PFS, indicating that patients who received pemetrexed‐based regimen were at a low risk of disease progression (HR  =  3.09, 95% CI 1.14–8.36, *p*  =  0.026) (Table  S1).

In the survival analysis, chemotherapy regimen, therapeutic efficacy, and the status of combination with bevacizumab were factors that affected the OS. Among them, the survival benefit of patients receiving GP regimen was significantly inferior to that of TP and PP regimens (17.5 vs. 24.6 vs. 26.8  months, HR  =  1.3, 95% CI 1.02–1.68, *p*  =  0.03 and HR  =  1.78, 95% CI 1.25–2.53, *p*  <  0.001, respectively) (Figure 3C). Chemotherapy in combination with bevacizumab was noted as another favorable factor affecting survival, with a median OS of not reached versus 23.5  months (HR  =  0.49, 95% CI 0.30–0.82, *p*  =  0.007; Figure 3D). Other factors, including sex, age, mutated sites of KRAS, and concomitant mutation were not correlated to the OS. The results of multivariate logistic regression analysis are listed in Table  S1, suggesting that no factor affected the OS of chemotherapy.

### 3.4 Response to ICIs and survival analysis

A total of 77 patients were treated with ICIs, of whom 45 underwent monotherapy with PD‐1/PD‐L1 antibody, 23 received concurrent chemotherapy, and nine received anti‐vascular therapy. Among the patients with definite efficacy (*n*  =  73), ORR and DCR were 26% (19/73) and 72.6% (53/73), respectively. Clinicopathological factors, including sex, smoking history, histology, and TNM stage did not significantly affect the therapeutic efficacy. Regarding mutated sites of KRAS, no significant differences were found in ORR and DCR between the G12X and G13X groups or among the most common three subtypes (33.3% in G12C, 28.6% in G12D, and 44.4% in G12V; *p*  =  0.96). Polygenic tests were performed on 51 patients, and similarly no significant efficacy differences were suggested when co‐mutations were concerned with ORR of 39.1% in patients with pure KRAS mutations, 8.3% in those harboring TP53 and 12.5% in those harboring STK11 (*p*  =  0.08). In terms of combination approach, a higher response rate was achieved in patients who were treated with chemotherapy simultaneously, compared with monotherapy and anti‐vascular therapy (40.9% vs. 21.4% vs. 11.1%, *p*  =  0.053) (Table 3).

**TABLE 3:** The association between clinicopathological factors and efficacy of immunotherapy

Characteristics	ORR (%)	DCR (%)	*p*‐value
Sex			0.066
Male	33.3 (17/51)	72.5 (47/51)	
Female	9.1 (2/22)	72.7 (16/22)	
Histology			0.20
Lung adenocarcinoma	22.6 (14/62)	71.0 (44/62)	
Squamous cell lung cancer	20 (1/5)	80 (4/5)	
Others	66.7 (4/6)	83.3 (5/6)	
Smoking history			0.61
Current/former	22.5 (9/40)	75.0 (30/40)	
No	28.1 (9/32)	68.7 (22/32)	
Number of metastatic sites			0.059
≥3	7.1 (1/14)	50 (7/14)	
<3	30.5 (18/59)	78 (46/59)	
Mutated sites of KRAS			0.96
G12C	33.3 (10/30)	70.0 (21/30)	
G12D	28.6 (2/7)	71.4 (5/7)	
G12V	44.4 (4/9)	77.8 (7/9)	
Co‐mutations			0.08
TP53	8.3 (1/12)	58.3 (7/12)	
STK11	12.5 (1/8)	87.5 (7/8)	
None	39.1 (9/23)	69.5 (16/23)	
Combination			0.053
Chemo	40.9 (9/22)	86.4 (19/22)	
Anti‐vascular agents	11.1 (1/9)	88.9 (8/9)	
None	21.4 (9/42)	61.9 (26/42)	

Abbreviations: DCR, disease control rate; KRAS, Kirsten rat sarcoma viral oncogene homolog; ORR, overall response rate; STK11, serine/threonine kinase 11; TP53, tumor protein p53.

**FIGURE 4:**
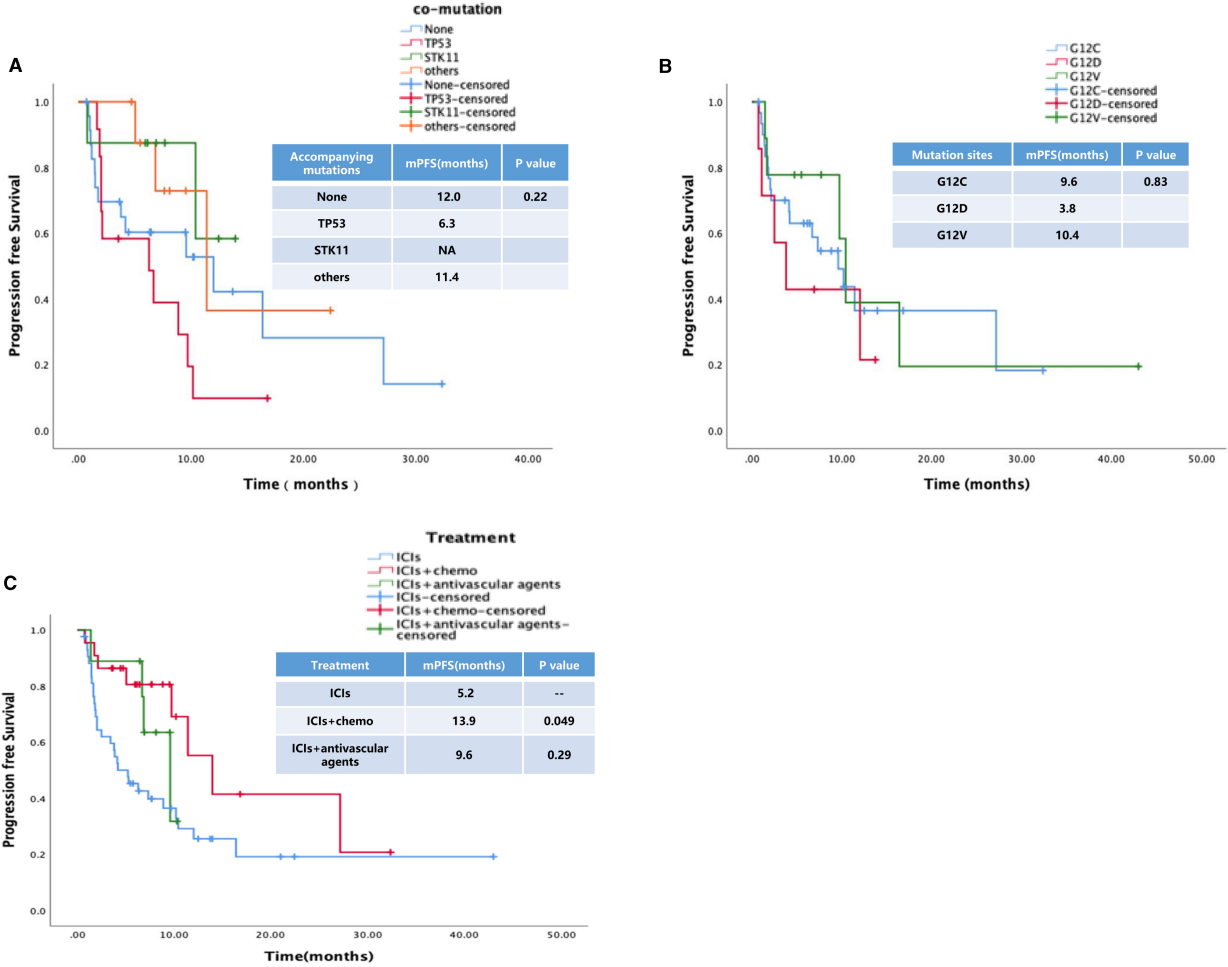
Survival analysis of patients with KRAS‐mutant NSCLC who were treated with ICIs. (A) PFS did not significantly differ between patients with or without accompanying mutations (TP53/STK11/others) (HR = 0.80, 95% CI 0.56–1.14, *p* = 0.22). (B) The median PFS of G12C/D/V was 9.6, 3.8, and 10.4 months, respectively (HR = 0.95, 95% CI 0.60–1.51, *p* = 0.83). (C) Concomitant chemotherapy significantly prolonged PFS compared with monotherapy (13.9 vs. 5.2 months, HR = 0.59, 95% CI 0.35–0.99, *p* = 0.049), while no significant difference was found compared with concomitant anti‐vascular therapy (9.6 vs. 5.2 months, HR = 0.53, 95% CI 0.18–1.53, *p* = 0.29). CI, confidence interval; HR, hazard ratio; ICI, immune checkpoint inhibitor; KRAS, Kirsten rat sarcoma viral oncogene homolog; NSCLC, non‐small cell lung cancer; PFS, progression‐free survival

The median PFS of patients with disease progression was 9.6  months (95% CI 6.22–12.97). Analyzing patients' survival data showed that no factor influenced PFS after immunotherapy (Table  S2). PFS did not differ between patients with or without accompanying mutations (HR  =  0.80, 95% CI 0.56–1.14, *p*  =  0.22) (Figure 4A). Regarding mutated sites of KRAS, there was no significant difference in PFS among the most common three mutational sites. The median PFS of G12C/D/V was 9.6, 3.8, and 10.4  months, respectively (HR  =  0.95, 95% CI 0.60–1.51, *p*  =  0.83; Figure 4B). In terms of combination therapy, concomitant chemotherapy significantly prolonged PFS compared with monotherapy (13.9 vs. 5.2  months, HR  =  0.59, 95% CI 0.35–0.99, *p*  =  0.049) (Figure 4C), while no significant difference was found between concomitant anti‐vascular therapy and monotherapy (9.6 vs. 5.2  months, HR  =  0.53, 95% CI 0.18–1.53, *p*  =  0.29). In addition, the results of multivariate analysis showed that there were also no factor influenced OS after immunotherapy (Table  S2).

## 4 DISCUSSION

In spite of a great progress achieved in KRAS‐mutant NSCLC, cytotoxic chemotherapy is still recommended as the standard treatment for patients with KRAS mutations. However, whether KRAS mutations could be evaluated as a predictive factor of chemotherapy remains controversial.^7^, ^8^, ^13^ In addition, KRAS subtypes may play an important role in clinical outcomes, including treatment response and PFS.^6^, ^7^, ^8^ Besides, immunotherapy (e.g., PD‐1/PD‐L1 antibody) is considered as an effective treatment for KRAS‐mutant NSCLC.^19^, ^20^, ^22^ However, few real‐world relevant studies have been conducted. The present retrospective study was undertaken to assess the clinicopathological features of patients with KRAS‐mutant NSCLC and to analyze the clinical outcomes of chemotherapy and immunotherapy.

In our study, as a KRAS study with the largest NGS sample size, KRAS mutation rate of NSCLC was 12.1% from the LANDSCAPE project and 11.4% in our center, consistent with or even higher than other studies on the Asian populations,^6^, ^23^ in which G12C mutations accounted for 30%–40%, indicating a large potential population for KRAS inhibitors. The therapeutic results revealed that the pemetrexed‐based regimen could be a factor related with prolonged PFS, even no significant difference was noted in the ORR. The potential efficacy of pemetrexed had been supported by a preclinical study in which greater dependency on folate metabolism pathways was identified in KRAS‐mutant NSCLC cell lines.^24^ Clinially, retrospective analysis also suggested the positive association between KRAS mutations and PFS of pemetrexed‐based regimen. In Lei's study, they found that pemetrexed‐based chemotherapy was superior to taxane based and gemcitabine‐based chemotherapies for patients with KRAS‐mutant NSCLC.^25^ However, other studies reported opposite findings. Mellema et al. found that PFS was significantly improved in patients treated with taxanes compared with pemetrexed.^8^ In another retrospective study on 1190 patients with KRAS‐mutant NSCLC performed by Massard et al., pemetrexed was associated with the worst time‐to‐progression (TTP) in the entire cohort.^26^ The discrepancy in the reported results could be related to the diverse chemotherapeutic regimens, sample size, etc. Thus, a randomized phase III study (NVALT22) aiming to compare the efficacy of “pemetrexed/cisplatin” and “paclitaxel/carboplatin/bevacizumab” in the first‐line treatment of patients with advanced KRAS‐mutant NSCLC was conducted, and showed that the two groups had a comparable PFS.^27^ However, the paclitaxel arm contained bevacizumab, a VEGF2 antibody which could improve clinical outcome of NSCLC. Therefore, the differences could not be directly compared between the two chemical agents.

Various studies have attempted to investigate the effects of different KRAS mutations on chemotherapy due to the heterogeneity of KRAS mutations, and controversial outcomes were reported. In an in vitro study conducted by Garassino et al., G12C mutation was associated with the increased sensitivity to taxol and pemetrexed. While G12D was associated with resistance to taxol and G12V showed a higher resistance to pemetrexed. The different therapeutic sensitivities might result from altered associations with downstream signaling pathways. Meanwhile, some previous studies reported a negative association of G12C/V with PFS.^6^, ^26^, ^28^ However, in real‐world study, no statistically significant difference was identified between the G12C and non‐G12C groups.^25^ The present study found no significant difference in the therapeutic efficacy based on amino acid substitutions. The existence of some objective factors could lead to inconsistent conclusions from the above‐mentioned studies, including the diversity of genetic‐based differences between Eastern and Western cases, chemotherapeutic regimens, sample size, etc. Despite the uncertainty, the detection of specific mutational sites is highly essential to select more appropriate individualized treatments.

In recent years, immunotherapy represented by PD‐1/PD‐L1 inhibitors has changed the treatment mode in advanced lung cancer, and has become the standard treatment option for NSCLC.^19^, ^29^, ^30^ Studies have shown that KRAS mutations are associated with a higher tumor mutational burden (TMB), upregulated PD‐L1 expression, and increased proportion of CD8+ tumor‐infiltrating lymphocytes (TILs), which could reflect an improved response to ICIs.^31^, ^32^ A three‐pool analysis including 1716 patients from nine studies was performed to assess the efficacy of PD‐1/PD‐L1 inhibitors, and showed significantly higher rates of ORR and 6‐month PFS in the KRAS‐mutant group.^33^ However, the other two studies reported inconsistent results, suggesting that KRAS mutational status does not associate with significant differences in ORR and PFS, which could be related to the heterogeneity of mutational sites and accompanying gene mutations (TP53 mutation or STK11/KEAP1 mutation).^34^, ^35^ In the present study, ORR of patients treated with ICIs was 26%, which is consistent with the previously reported rate.^35^ Moreover, we found no significant differences among subtypes of KRAS mutations, either monotherapy or combination therapy, which was consistent with Janson et al.’s findings.^34^ While data from Dana‐Farber Hospital presented in the ASCO meeting (2019) showed longer PFS (5.5 vs. 2.7  months, *p*  =  0.03) and OS (17.5 vs. 9.7  months, *p*  =  0.05) in G12V mutant patients compared with non‐G12V mutant cases.^36^ The advantage of G12V in response to immunotherapy could be attributed to a higher expression of PD‐L1, as reported by Falk et al. (12.9% TPS in G12V, 8% TPS in G12C, and 5.3% TPS in G12D, *p*  =  0.044).^37^ The discrepancy in the above‐mentioned studies is likely due to the heterogeneity of sample size, treatment lines, and accompanying gene mutations. In previous studies, concurrent pathogenic mutations in TP53/STK11/KEAP1 may be positive/negative factors of treatment efficacy and survival in KRAS‐mutant NSCLC patients. The mechanism could be illustrated by PD‐L1 expression, TMB, and the tumor immune microenvironment (CD8+ T‐cell infiltration).^38^, ^39^ In the present study, the presence of STK11 or TP53 mutations was not a factor leading to survival difference. Small sample size and inconsistent gene detection methods may be responsible for the controversial results. In addition, we found that the addition of chemotherapy could significantly improve the efficacy of ICIs, including first‐line and posterior lines of therapy. However, due to the small sample size, the expressions of PD‐L1 and accompanying mutations were not considered, which might lead to a statistical bias, the relationship between combination modalities and OS still remains uncertain. Real‐world studies with a larger sample size and stricter inclusion criteria are needed to further confirm our results.

Notably, three patients with G12C mutations in our study have been treated with KRAS inhibitors (JAB‐21822, from phase I clinical trials) and achieved disease remission. In real‐world clinical environment, the attractive results of sotorasib(AMG‐510) make it the first approved drug to treat KRAS (G12C)‐mutant NSCLC in US. In CodeBreak 100 study, the therapeutic effect of sotorasib was also assessed with PD‐L1 expression, and the potential association with STK11, KEAP1, and TP53 mutations. However, even if STK11 mutant/KEAP1 wild‐type patients responsed better than others, future prospective studies are warranted to identify the ones who may benefit differently from sotorasib due to small subgroup sample sizes in this trial.^40^ Meanwhile, phase III trial CodeBreak 200 (NCT04303780), comparing sotorasib with docetaxel, as well as different clinical trials investigating combination therapies (CodeBreaK101; NCT04185883) are ongoing with the aim to identify more patients who may benefit from sotorasib regimens.

This was the first study to analyze the treatment of KRAS‐mutant NSCLC patients in China, including chemotherapy and immunotherapy. However, several limitations of this study should be acknowledged. First, the retrospective design might lead to bias. In addition, genetic testing methods were not uniform. NGS, PCR, and DHPLC with different sensitivities were all included, resulting in severely censored clinical data, such as mutational sites and concurrent mutations, and the corresponding conclusions could not be drawn. Furthermore, a large time span in the study might affect the uniformity of treatment regimens, including selection of the chemotherapeutic drugs, combination with bevacizumab, and maintenance therapy. Moreover, in the immunotherapy population, a small sample size and lack of PD‐L1 expression and TMB data might result in bias of immunotherapy results. Thus, additional studies with more uniform protocols are needed to answer the questions we currently encounter (i.e., which chemotherapeutic drugs and immunotherapy regimens are more appropriate for the KRAS‐mutant NSCLC patients).

## 5 CONCLUSIONS

Overall, the rate of KRAS mutation was approximately 11.4%–12.1% in the Chinese patients with KRAS‐mutant NSCLC, and G12C was found as the most common subtype. In chemotherapy, patients with KRAS mutations could benefit more from the pemetrexed‐based regimen, regardless of mutational sites. ICIs may be a fruitful treatment modality for patients with KRAS‐mutant NSCLC, especially combined with chemotherapy.

### CONTRIBUTION TO THE FIELD STATEMENT

KRAS mutation is a relatively specific subtype in NSCLC because of a poor accessibility to targeted agents and a minor response to chemotherapy. In the present study, we retrospectively collected the epidemiological data of KRAS mutations from 150,327 NGS cases, providing a solid data basis for the application of KRAS inhibitors in the future. In addition, the present study also analyzed the discrepancy in efficacy of different chemotherapeutic regimens, suggesting a superior benefit of pemetrexed combined regimen, regardless of mutation subtypes. Alternatively, in terms of immunotherapy, we found that KRAS mutant patients benefit from ICIs, regardless of molecular subtype. All the findings of this study may provide a reliable basis for the treatment of patients with KRAS‐mutant NSCLC.

### ETHICAL APPROVAL STATEMENT

The study was approved by the Ethics Committee of Beijing Cancer Hospital (Beijing, China; Approval No. 2021KT33).

## CONFLICT OF INTEREST

The authors declare no potential conflict of interest.

## AUTHOR CONTRIBUTIONS

Hanxiao Chen and Dingzhi Huang collected the samples and clinical data. Hanxiao Chen wrote the manuscript. Gen Lin, Xue Yang, Minglei Zhuo, Yujia Chi, Xiaoyu Zhai, Bo Jia, Jingjing Wang, Yuyan Wang, Jianjie Li, Tongtong An, Meina Wu, and Ziping Wang contributed in patient caring. Jun Zhao designed and regulated this study.

## Supporting information


Table S1

Table S2

## Data Availability

The data that support the findings of this study are available from the corresponding author upon reasonable request.
