# Supplementary material for: The prevalence and real‐world therapeutic analysis of Chinese patients with KRAS‐Mutant Non‐Small Cell lung cancer
Source: Cancer Med. 2022 Apr 8;11(19):3581–92. doi: 10.1002/cam4.4739 (PMC9554448; doi:10.1002/cam4.4739)
Supplement: Supplementary file 1 — Table S1 [file CAM4-11-3581-s002.docx]

Supplementary Table 1 Results of univariate and multivariate logistic regression analyses of PFS (1-st chemo) and OS in patients with KRAS-mutant NSCLC.

| Characteristics | PFS | | | | | | OS | | | | | |
| --- | --- | --- | --- | --- | --- | --- | --- | --- | --- | --- | --- | --- |
|  | Univariate analysis | | | Multivariate analysis | | | Univariate analysis | | | Multivariate analysis | | |
|  | HR | 95%CI | *P*-value | HR | 95%CI | *P*-value | HR | 95%CI | *P*-value | HR | 95%CI | *P*-value |
| Sex | 0.87 | 0.65-1.17 | 0.36 | 2.57 | 0.43-15.43 | 0.30 | 0.75 | 0.53-1.06 | 0.10 | 4.40 | 0.54-36.1 | 0.17 |
| Histology | 1.25 | 0.99-1.57 | 0.06 | 1.03 | 0.33-1.47 | 0.34 | 1.19 | 0.88-1.61 | 0.27 | 0.55 | 0.11-2.69 | 0.46 |
| Smoking history | 0.97 | 0.75-1.26 | 0.81 | 2.03 | 0.38-10.96 | 0.41 | 1.08 | 0.79-1.48 | 0.62 | 4.70 | 0.00-5.79 | 0.94 |
| KRAS subtype | 1.09 | 0.86-1.39 | 0.49 | 1.13 | 0.73-1.29 | 0.73 | 1.06 | 0.74-1.51 | 0.75 | 1.78 | 0.55-5.68 | 0.33 |
| Chemo regimens | 1.18 | 0.95-1.46 | 0.12 | 3.09 | 1.14-8.36 | **0.026** | 1.30 | 1.04-1.61 | **0.02** | 5.23 | 0.76-36.28 | 0.09 |
| Bevacizumab combined | 0.82 | 0.59-1.13 | 0.22 | 0.98 | 0.37-2.63 | 0.97 | 0.49 | 0.30-0.82 | **0.007** | 0.29 | 0.04-2.05 | 0.22 |

CI: confidence interval; HR: hazard ratio; KRAS: kirsten rat sarcoma viral oncogene homolog; NSCLC: non-small-cell lung cancer; OS: overall survival; PFS: progression-free survival.
