# Supplementary material for: The prevalence and real‐world therapeutic analysis of Chinese patients with KRAS‐Mutant Non‐Small Cell lung cancer
Source: Cancer Med. 2022 Apr 8;11(19):3581–92. doi: 10.1002/cam4.4739 (PMC9554448; doi:10.1002/cam4.4739)
Supplement: Supplementary file 2 — Table S2 [file CAM4-11-3581-s001.docx]

Supplementary Table 2 Results of univariate and multivariate logistic regression analyses of PFS and OS of ICIs in patients with KRAS-mutant NSCLC.

| Characteristics | PFS | | | | | | OS | | | | | |
| --- | --- | --- | --- | --- | --- | --- | --- | --- | --- | --- | --- | --- |
|  | Univariate analysis | | | Multivariate analysis | | | Univariate analysis | | | Multivariate analysis | | |
|  | HR | 95%CI | *P*-value | HR | 95%CI | *P*-value | HR | 95%CI | *P*-value | HR | 95%CI | *P*-value |
| Sex | 0.54 | 0.29-1.04 | 0.064 | 0.70 | 0.06-8.00 | 0.77 | 1.16 | 0.43-3.08 | 0.77 | 2.28 | 0.15-35.4 | 0.56 |
| Histology | 0.99 | 0.56-1.77 | 0.98 | 0.45 | 0.08-2.58 | 0.37 | 1.07 | 0.48-2.40 | 0.87 | 1.89 | 0.19-18.7 | 0.59 |
| KRAS subtype | 0.95 | 0.60-1.51 | 0.83 | 4.23 | 0.89-20.67 | 0.07 | 1.12 | 0.58-2.14 | 0.74 | 1.75 | 0.16-19.06 | 0.64 |
| Accompanying mutation | 0.80 | 0.56-1.14 | 0.22 | 2.26 | 0.09-50.5 | 0.75 | 0.39 | 0.15-1.08 | 0.06 | 0.16 | 0.02-1.29 | 0.086 |
| Combination approach | 0.59 | 0.35-0.99 | **0.049** | 0.27 | 0.02-3.59 | 0.32 | 2.62 | 0.08-0.81 | **0.02** | 0.76 | 0.03-17.2 | 0.86 |

CI: confidence interval; HR: hazard ratio; ICI: immune checkpoint inhibitors; KRAS: kirsten rat sarcoma viral oncogene homolog; NSCLC: non-small-cell lung cancer; OS: overall survival;PFS: progression-free survival.
